# ORBIT‐AMD: Ordinal Risk, Bilateral Imaging, and Trajectory Learning for Age‐Related Macular Degeneration in Multi‐Cohorts

**DOI:** 10.1002/advs.76361

**Published:** 2026-06-27

**Authors:** Xuehao Cui, Dejia Wen, Patrick Yu‐Wai‐Man, Xiaorong Li

**Affiliations:** ^1^ Eye Institute and School of Optometry Tianjin Medical University Eye Hospital Tianjin China; ^2^ Tianjin Key Laboratory of Retinal Functions and Diseases Tianjin China; ^3^ John Van Geest Centre for Brain Repair and MRC Mitochondrial Biology Unit Department of Clinical Neurosciences University of Cambridge Cambridge UK; ^4^ Cambridge Eye Unit Addenbrooke's Hospital Cambridge University Hospitals Cambridge UK

**Keywords:** age‐related macular degeneration, artificial intelligence, disease progression, interpretable machine learning, optical coherence tomography, retinal imaging

## Abstract

Age‐related macular degeneration (AMD) is an ordered, bilateral, and longitudinal disease, yet many artificial intelligence systems treat it as static binary image classification. We developed ORBIT‐AMD, a multimodal trajectory‐learning framework integrating color fundus photography and optical coherence tomography, bilateral eye‐graph attention, concept bottlenecks, ordinal staging, cause‐specific discrete‐time survival prediction, and protocol alignment. In a UK Biobank development/internal‐testing cohort of 58 214 participants and 109 691 eyes, and an external Tianjin Medical University Eye Hospital cohort of 1996 participants and 3780 eyes, ORBIT‐AMD achieved AUROC values of 0.984 internally and 0.975 externally for prevalent late‐AMD detection. Five‐year late‐AMD progression prediction achieved AUROC values of 0.825 and 0.767, respectively. Calibration and threshold analyses showed cohort‐dependent workload and absolute‐risk behavior, supporting site‐specific calibration assessment and clinical‐workflow evaluation before deployment. The concept bottleneck provided auditable lesion‐level explanations, but these outputs should be interpreted as structured predictive explanations rather than causal evidence. ORBIT‐AMD provides a trajectory‐aware framework for AMD risk stratification and review prioritization, with prospective validation required before clinical implementation.

## Graphical abstract

Eligibility flow and real‐world AMD burden in the UKB retinal imaging cohort and TMUEH external‐validation cohort. Overview of the ORBIT‐AMD architecture, integrating retinal representation pretraining, bilateral eye‐graph modeling and concept bottleneck learning to support ordered risk, bilateral context, interpretable lesion concepts, longitudinal hazards and protocol alignment. Participant‐level UKB training, validation and internal‐test split, with the full TMUEH cohort retained as an independent external test set. Endpoint and event counts across cohorts, including baseline late AMD, incident late AMD by 5 years, and eligible eyes. AMD, age‐related macular degeneration; UKB, UK Biobank; TMUEH, Tianjin Medical University Eye Hospital.

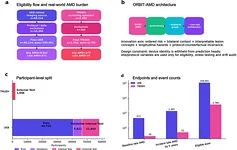

## 1 Introduction

Age‐related macular degeneration (AMD) is a progressive retinal disease and one of the leading causes of irreversible central vision loss in older adults [1]. Its clinical importance arises not only from the presence of late disease, but also from the prolonged and heterogeneous transition from early drusen and pigmentary change to intermediate AMD, geographic atrophy (GA) or neovascular AMD (nAMD) [2, 3, 4, 5]. In population screening, the key clinical question is whether a participant already shows early or intermediate AMD requiring surveillance [6, 7]. In retina clinics, the more actionable question is whether a patient with drusen burden, pigmentary abnormality, hyperreflective foci, ellipsoid‐zone disruption, subretinal drusenoid deposits, or fellow‐eye involvement will progress to late AMD within a clinically meaningful time window [8, 9, 10, 11, 12].

Machine learning has improved automated analysis of retinal images, including disease classification, lesion detection, risk prediction, and triage support [13]. Retinal foundation models and self‐supervised learning have further shown that transferable image representations can be extracted from large retinal image collections [14]. However, AMD remains a challenging target for artificial intelligence because it is ordered, bilateral, longitudinal, and biologically heterogeneous [15, 16]. A model that detects prevalent late AMD with high discrimination may still be insufficient for surveillance planning if it does not represent disease stage, future transition risk, absolute calibration, uncertainty, or clinical workload [15, 17, 18].

Several gaps limit current AMD machine‐learning studies. First, many models collapse AMD into a binary endpoint, losing the ordinal structure used by ophthalmologists when assessing drusen size, pigmentary abnormality, atrophy, and exudation [19]. Second, validation is often internal or weakly external, which can overestimate transportability when image acquisition, label style, and disease prevalence are similar across datasets. Third, device, camera style, and acquisition protocol may act as hidden shortcuts. A model may learn image texture, signal‐to‐noise pattern, or cohort prevalence rather than AMD biology [20]. Fourth, many reports focus on area under the receiver operating characteristic curve (AUROC) but underreport calibration, decision thresholds, workload consequences, subgroup behavior, and failure modes, although these quantities determine whether a model can be used safely [21].

Another important limitation is interpretability and auditability. Ophthalmologists do not reason only from a black‐box probability. They interpret disease through clinically meaningful lesion concepts, including drusen area, drusen volume, pigment disturbance, hyperreflective foci, ellipsoid‐zone integrity, GA, exudative fluid, and hemorrhage [22]. A high‐risk prediction driven by drusen burden and ellipsoid‐zone disruption has different clinical implications from one dominated by poor image quality or cohort‐specific acquisition style. Therefore, a clinically useful AMD model should expose the lesion‐related explanatory pattern behind its prediction while avoiding unsupported causal interpretation.

We designed ORBIT‐AMD to model AMD as a disease trajectory rather than a static label. The framework integrates masked bimodal retinal representation learning from color fundus photography (CFP) and optical coherence tomography (OCT), bilateral eye‐graph attention for fellow‐eye asymmetry, an auditable concept bottleneck, joint ordinal staging and cause‐specific discrete‐time survival prediction, and counterfactual protocol alignment to reduce acquisition‐style shortcuts [23, 24]. Device identity was not used as a prediction feature, while protocol information was retained for eligibility assessment, sensitivity analysis, and drift audit. Using UK Biobank (UKB) as the development and internal‐testing cohort and Tianjin Medical University Eye Hospital (TMUEH) as the independent external‐validation cohort, this real‐world multi‐cohort study evaluated discrimination, calibration, threshold‐dependent workload, label‐source robustness, and interpretability under clinically relevant cohort shift.

## 2 Results

### 2.1 Cohort Characteristics and Endpoints

The analysis included 58 214 UKB participants with 109 691 eligible eyes and 1996 TMUEH external‐validation participants with 3780 eligible eyes (Figure 1a and Table S1). Sequential eligibility checks reduced the UKB source imaging pool from 68 514 to 58 214 participants after exclusions for missing paired or core modality information, ungradable or nonprotocol‐locked imaging, non‐AMD macular comorbidity, missing core covariates or follow‐up, and linkage‐, visit‐level‐ or protocol‐harmonisation‐related ineligibility (Table S1). The UKB cohort was then split at the participant level into 40 750 training, 5821 validation and 11 643 internal‐test participants using stage‐aware stratification, while the full TMUEH cohort was retained as the independent external test set (Figure 1c and Table S13). UKB participants were younger than TMUEH participants (mean age 57.0 years, SD 7.4 vs. 66.8 years, SD 8.6), whereas TMUEH participants had higher hypertension and diabetes prevalence, consistent with an older hospital‐based screening and routine‐clinic population (Table 1; Figure 2a). In UKB, 49,272 participants had no AMD, 6348 had early AMD, 2123 had intermediate AMD, and 471 had late AMD; in TMUEH, the corresponding numbers were 1717, 190, 60, and 29. Any AMD prevalence was 15.4% in UKB and 14.0% in TMUEH, while late AMD prevalence was 0.81% and 1.45%, respectively (Figure 2b). Among baseline non‐late participants, 5‐year incident late AMD occurred in 1387 UKB participants and 52 TMUEH participants, with median follow‐up of 10.2 and 4.1 years, respectively (Figure 1d). Baseline late‐AMD cases were distributed across UKB training, validation, and internal‐test sets as 334, 47, and 90 participants, respectively, and 5‐year incident late‐AMD events among baseline nonlate participants were 966, 139, and 282, respectively (Table S13). These event counts provide the basis for interpreting progression performance, calibration, and threshold‐dependent workload under realistic external event scarcity.

**FIGURE 1:**
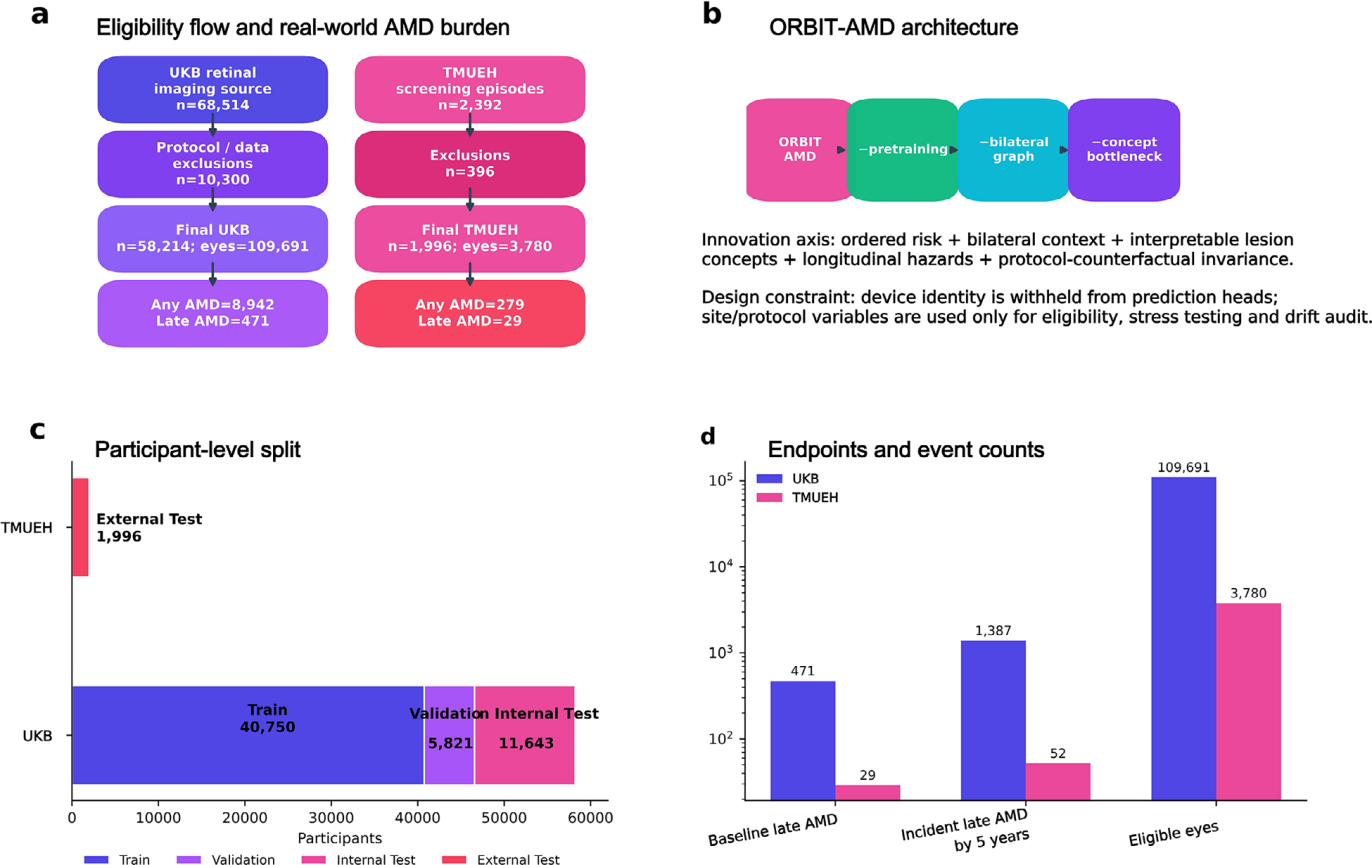
Study design, cohort scale and ORBIT‐AMD model blueprint. (a) Eligibility flow and real‐world AMD burden in the UKB retinal imaging cohort and TMUEH external‐validation cohort. (b) Overview of the ORBIT‐AMD architecture, integrating retinal representation pretraining, bilateral eye‐graph modeling and concept bottleneck learning to support ordered risk, bilateral context, interpretable lesion concepts, longitudinal hazards and protocol alignment. (c) Participant‐level UKB training, validation and internal‐test split, with the full TMUEH cohort retained as an independent external test set. (d) Endpoint and event counts across cohorts, including baseline late AMD, incident late AMD by 5 years, and eligible eyes. AMD, age‐related macular degeneration; UKB, UK Biobank; TMUEH, Tianjin Medical University Eye Hospital.

**TABLE 1:** Baseline characteristics of multi cohorts.

Characteristic	Public Cohort	TMUEH Cohort
Participants, n	58,214	1,996
Eligible eyes, n	109,691	3,780
Age, mean (SD), years	57.0 (7.4)	66.8 (8.6)
Female sex, n (%)	30,885 (53.1)	1,132 (56.7)
Diabetes, n (%)	2,389 (4.1)	183 (9.2)
Hypertension, n (%)	10,182 (17.5)	714 (35.8)
Current smoking, n (%)	7,214 (12.4)	299 (15.0)
No AMD, n (%)	49,272 (84.6)	1,717 (86.0)
Early AMD, n (%)	6,348 (10.9)	190 (9.5)
Intermediate AMD, n (%)	2,123 (3.6)	60 (3.0)
Late AMD, n (%)	471 (0.81)	29 (1.45)
Any AMD, n (%)	8,942 (15.4)	279 (14.0)
Incident late AMD by 5 years among baseline non‐late, n (%)	1,387 (2.4)	52 (2.6)
Follow‐up, median (IQR), years	10.2 (9.7–10.7)	4.1 (3.2–5.2)
Mean CFP quality (SD)	0.912 (0.048)	0.878 (0.050)
Mean OCT quality (SD)	0.931 (0.041)	0.897 (0.042)

Baseline demographic, clinical, AMD grading, follow‐up, and imaging quality characteristics are summarised for the Public Cohort (UKB) and TMUEH Cohort. Data are presented as mean (SD), median (IQR), or n (%), as appropriate.

**FIGURE 2:**
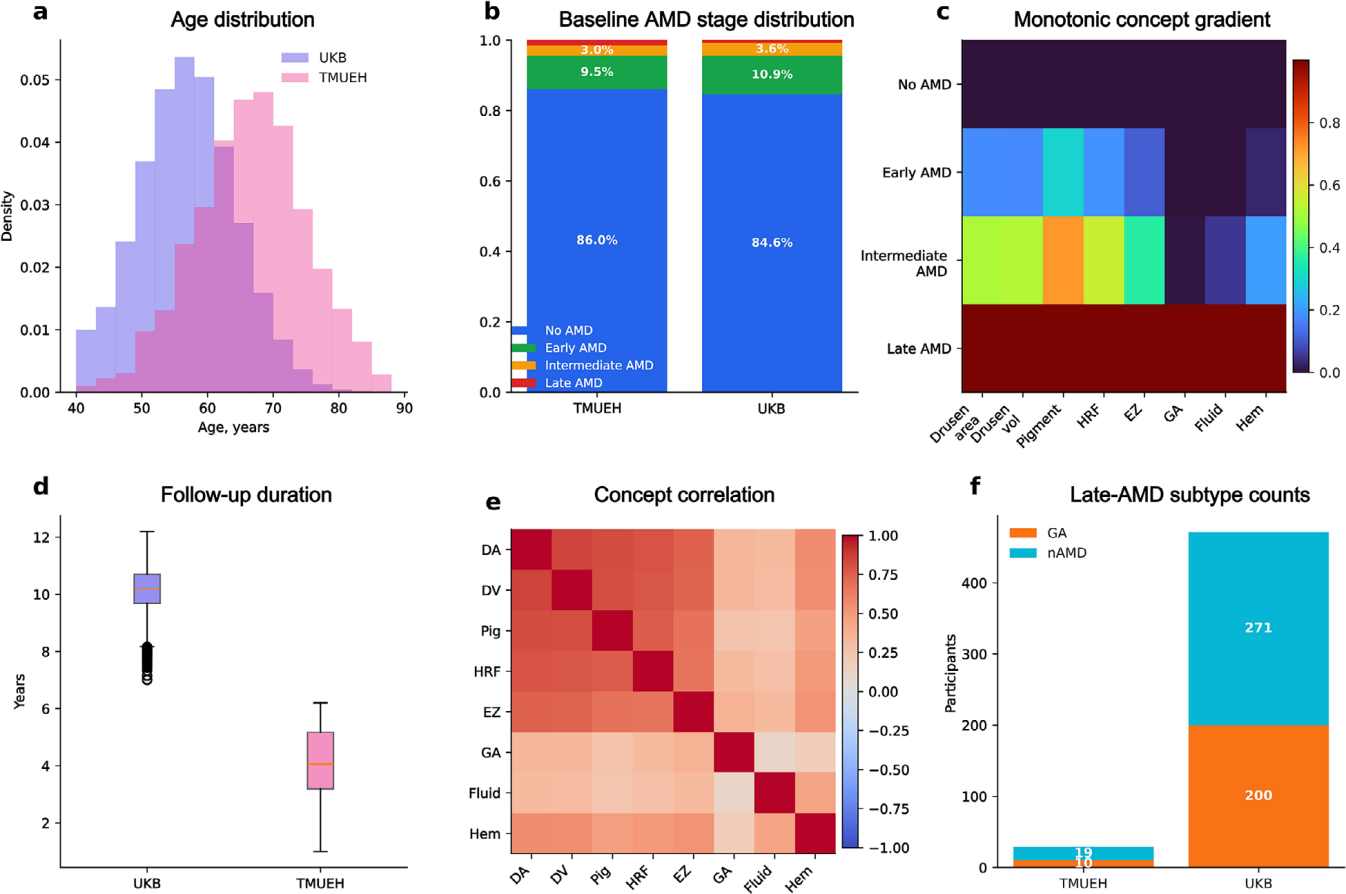
Cohort Characteristics, AMD Prevalence and Lesion‐concept Structure. (a) Age distributions in the UKB and TMUEH cohorts. (b) Baseline AMD stage distribution by cohort. (c) Stage‐conditioned gradient of image‐derived AMD concept estimates, including drusen area, drusen volume, pigment disturbance, hyperreflective foci, ellipsoid‐zone disruption, geographic atrophy, exudative fluid, and hemorrhage. (d) Follow‐up duration by cohort. (e) Correlation structure among lesion‐concept estimates. (f) Counts of late‐AMD subtypes, including geographic atrophy and neovascular AMD. AMD, age‐related macular degeneration; GA, geographic atrophy; nAMD, neovascular AMD; HRF, hyperreflective foci; EZ, ellipsoid zone; DA, drusen area; DV, drusen volume; Pig, pigment disturbance; Hem, hemorrhage.

### 2.2 Disease Concepts Followed a Clinically Plausible AMD Gradient

Image‐derived concept estimates showed ordered and clinically plausible trends across AMD stage (Figure 2c). Drusen area and drusen volume increased from no AMD through early and intermediate AMD, whereas GA area and exudative fluid volume were concentrated in late AMD. Pigment disturbance, hyperreflective foci, and ellipsoid‐zone disruption occupied an intermediate position: uncommon in no AMD, more frequent in early and intermediate AMD, and stronger in late AMD.

Concept correlations were positive but not redundant (Figure 2e). Drusen area correlated strongly with drusen volume and pigment disturbance, whereas correlations between drusen burden and late atrophic or exudative lesions were weaker. This structure created a clinically realistic learning problem, requiring the model to learn a shared early/intermediate substrate while separating atrophic and neovascular late pathways. Image quality was lower in TMUEH participants and in more severe disease (Figure S1 and Table S3), but image quality was used for eligibility assessment and audit rather than as a direct disease‐prediction feature.

### 2.3 ORBIT‐AMD Learned an Ordered, Concept‐Informed Disease Manifold

ORBIT‐AMD combined a masked bimodal encoder, bilateral eye graph, concept bottleneck, ordinal stage head, and discrete‐time survival head (Figure 1b). In latent projection, no AMD, early AMD, intermediate AMD, and late AMD formed a continuous severity manifold rather than four isolated clusters (Figure 3a). This geometry is clinically meaningful because most misclassifications occurred near neighboring stage boundaries, and prediction uncertainty was highest around early/intermediate transition zones (Figure 3d,f).

**FIGURE 3:**
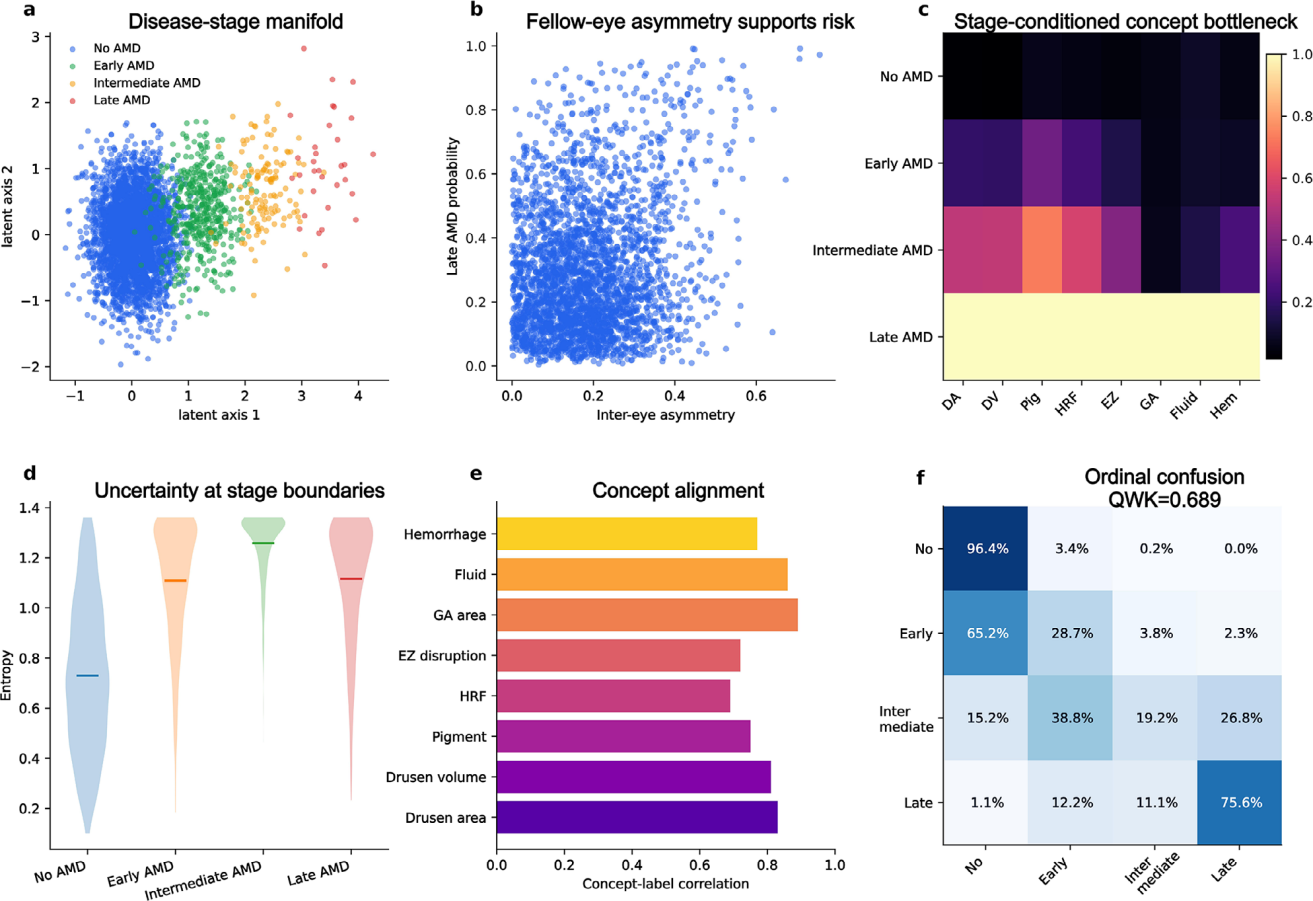
ORBIT‐AMD Learns an Ordered, Concept‐informed AMD Trajectory. (a) Latent Disease‐stage Manifold Showing the Ordered Distribution of no AMD, Early AMD, Intermediate AMD, and Late AMD. (b) Relationship Between Inter‐eye Asymmetry and Predicted Late‐AMD Probability. (c) Stage‐conditioned Concept Bottleneck Values Across AMD Stages. (d) Prediction Uncertainty Across Stage Boundaries. (e) Concept‐label Alignment for Individual Lesion Concepts. (f) Row‐normalized Ordinal Confusion Matrix for Four‐stage AMD Classification in the UKB Internal Test Set. Labels Are Shown Consistently as no AMD, Early AMD, Intermediate AMD, and Late AMD. AMD, Age‐related Macular Degeneration; HRF, Hyperreflective Foci; EZ, Ellipsoid Zone; GA, Geographic Atrophy; QWK, Quadratic Weighted Kappa.

Fellow‐eye asymmetry contributed to risk estimation. Participants with greater inter‐eye asymmetry had higher late‐AMD probability, particularly when one eye carried intermediate disease or latent late‐lesion concepts (Figure 3b). The concept bottleneck remained interpretable: drusen and pigment concepts increased across early and intermediate stages, whereas GA and fluid were concentrated in late AMD (Figure 3c). Concept‐label correlations ranged from 0.68 to 0.88, with GA and fluid showing the strongest alignment and hyperreflective foci showing greater biological ambiguity (Figure 3e).

Ordinal staging preserved the ordered structure of AMD severity, although the exact four‐stage classification remained limited for early and intermediate stages. In the UKB internal test set, four‐stage accuracy was 0.863, macro‐F1 was 0.504, and quadratic weighted kappa was 0.689. In TMUEH external testing, the corresponding values were 0.859, 0.561, and 0.738 (Figure 3f and Table S5). Row‐normalized analysis of the UKB confusion matrix showed high recall for no AMD (96.4%) and late AMD (75.6%), but lower recall for early AMD (28.7%) and intermediate AMD (19.2%). Most errors were adjacent‐stage or boundary‐zone errors rather than extreme no‐AMD to late‐AMD disagreements, supporting the use of quadratic weighted kappa and adjacent‐stage accuracy as clinically relevant ordinal metrics (Table S15). These findings indicate that ORBIT‐AMD is better suited to risk stratification and review prioritization than to replacing specialist four‐stage AMD grading, particularly for early/intermediate boundary cases.

### 2.4 Late‐AMD Detection Retained High Internal and External Discrimination

For prevalent late‐AMD detection, ORBIT‐AMD achieved internal‐test AUROC 0.984, 95% confidence interval (CI) 0.977–0.992, and area under the precision‐recall curve (AUPRC) 0.619, 95% CI 0.530–0.706 (Figure 4a,d and Table S5). In TMUEH external testing, AUROC was 0.975, 95% CI 0.952–0.989, and AUPRC was 0.520, 95% CI 0.408–0.731 (Figure 4b,e and Table S5). The external confidence interval was wider because only 29 baseline late‐AMD cases were present, but the point estimate remained high.

**FIGURE 4:**
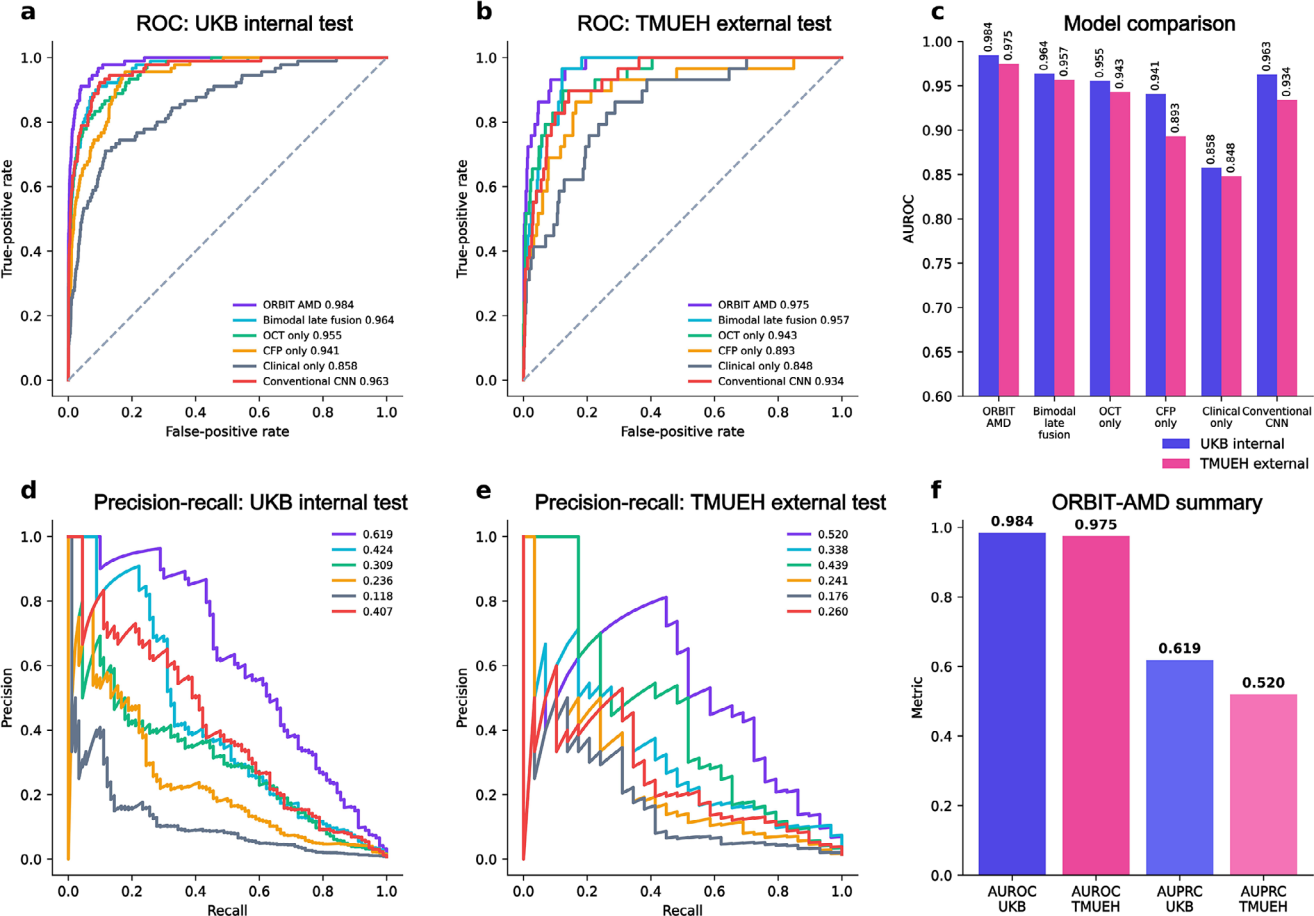
Prevalent Late‐AMD Detection Across Internal and External Cohorts. (a, b) Receiver operating characteristic curves for prevalent late‐AMD detection in the UKB internal test set and TMUEH external test set. (c) AUROC comparison between ORBIT‐AMD and baseline models. (d, e) Precision‐recall curves in the UKB internal and TMUEH external test sets. (f) Summary of ORBIT‐AMD AUROC and AUPRC across cohorts. AUROC, area under the receiver operating characteristic curve; AUPRC, area under the precision‐recall curve; CFP, color fundus photography; OCT, optical coherence tomography; CNN, convolutional neural network; AMD, age‐related macular degeneration.

Comparative models were consistently weaker (Figure 4c). The bimodal late‐fusion model reached AUROC 0.964 internally and 0.957 externally. OCT‐only achieved 0.955 and 0.946, CFP‐only achieved 0.941 and 0.893, clinical‐only achieved 0.858 and 0.848, and a conventional convolutional neural network achieved 0.963 and 0.934 (Figure S4). The gain over CFP‐only was especially important externally, suggesting that OCT‐derived structure and concept‐trajectory learning recovered information not captured by CFP alone.

Precision‐recall behavior reflected the low prevalence of late AMD in the external cohort. Although external AUROC remained high, external AUPRC was 0.520 because late AMD represented only 1.45% of the TMUEH cohort. This result emphasizes that AUROC alone is insufficient for evaluating clinical deployment: in low‐prevalence settings, threshold‐specific sensitivity, specificity, PPV, false‐positive burden, and missed‐case review are required to judge practical utility.

### 2.5 Five‐Year Progression Prediction, Calibration and Decision Consequences

Among baseline non‐late participants, ORBIT‐AMD predicted 5‐year late‐AMD progression with internal AUROC 0.825, 95% CI 0.804–0.854, and AUPRC 0.179, 95% CI 0.149–0.216. External AUROC was 0.767, 95% CI 0.715–0.821, and AUPRC was 0.078, 95% CI 0.053–0.131 (Figure 5a–c and Table S5). These progression estimates were generated by a cause‐specific discrete‐time survival head; death, loss to follow‐up, and administrative end of follow‐up were treated as censoring rather than as explicit competing‐risk events (Table S14). Discrimination was lower than for prevalent late‐AMD detection, as expected for a longitudinal event influenced by follow‐up intensity, treatment exposure, endpoint ascertainment, competing mortality, censoring, and biological variability. A quantitative cohort‐ and label‐source audit showed that the larger internal‐to‐external performance reduction for 5‐year progression prediction coincided with older external age distribution, shorter follow‐up, fewer progression events, and different endpoint provenance, whereas prevalent late‐AMD detection transported more strongly across cohorts (Table S18). These findings suggest that longitudinal risk prediction is more sensitive than baseline late‐AMD detection to follow‐up structure, event scarcity, and endpoint‐adjudication differences.

**FIGURE 5:**
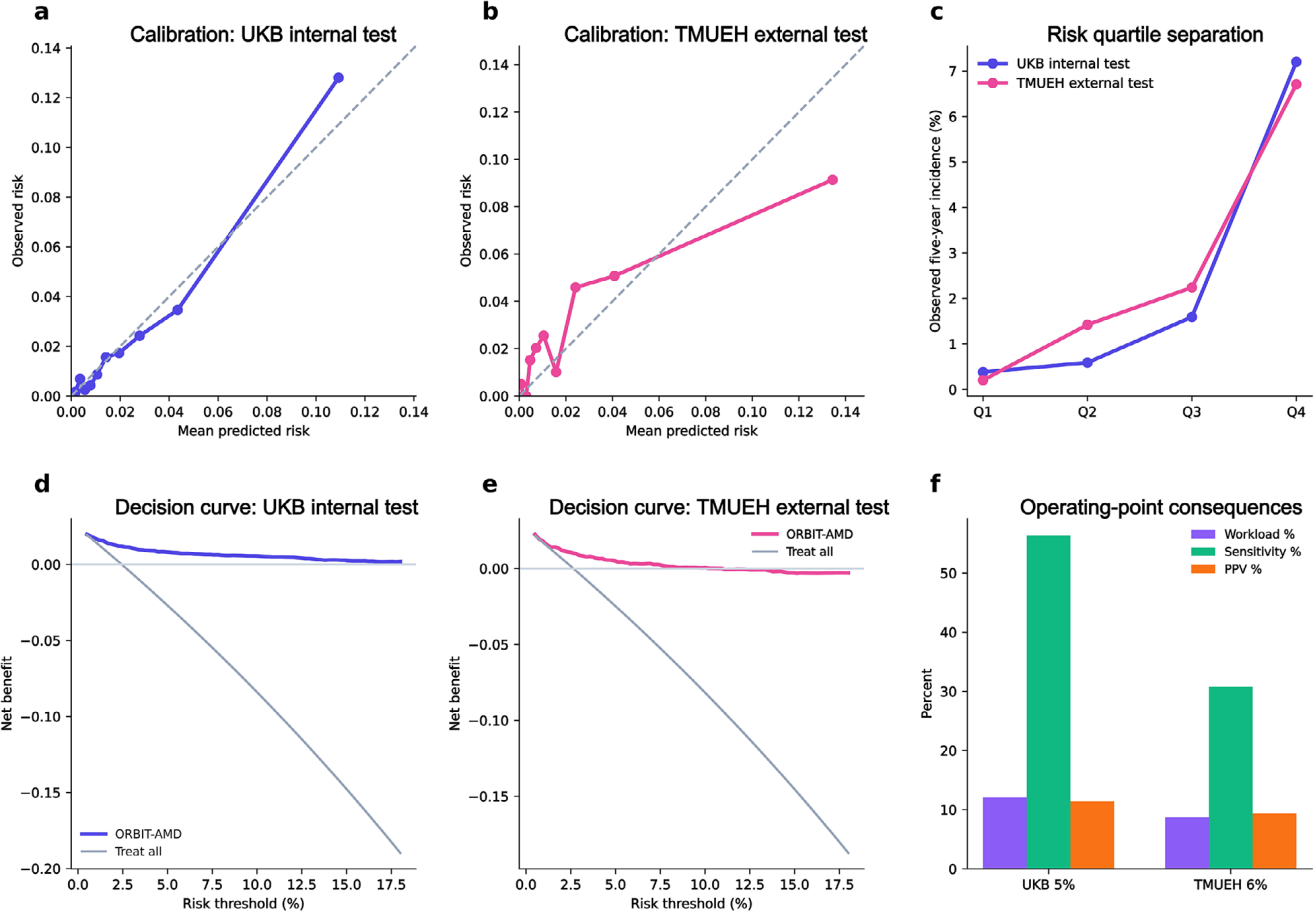
Five‐year Late‐AMD Progression Prediction, Calibration and Decision Consequences. (a, b) Calibration curves for 5‐year late‐AMD progression risk in the UKB internal test set and TMUEH external test set. (c) Observed 5‐year progression incidence across predicted‐risk quartiles. (d, e) Decision‐curve analysis in the UKB internal and TMUEH external test sets. (f) Threshold‐specific workload, sensitivity and positive predictive value at the selected UKB and TMUEH operating points. The TMUEH 6% operating point represents a low‐workload review‐prioritization threshold rather than a high‐sensitivity screening threshold. Progression estimates were generated using a cause‐specific discrete‐time survival formulation and should not be interpreted as competing‐risk cumulative incidence. Calibration and decision‐curve estimates should be interpreted in light of the smaller number of external progression events. AMD, age‐related macular degeneration; PPV, positive predictive value; UKB, UK Biobank; TMUEH, Tianjin Medical University Eye Hospital.

Calibration was acceptable in the low‐to‐intermediate predicted‐risk range but became less stable in the highest‐risk bins (Figure 5a, Figure S5, and Table S9). The external calibration curve showed greater uncertainty because the TMUEH cohort had fewer progression events and a shorter median follow‐up (Figure 5b). External expected calibration error (ECE) for progression was 0.0128 before local recalibration. A 300‐case Platt calibration, quality‐aware recalibration, and uncertainty routing reduced ECE to 0.0089, 0.0079, and 0.0071, respectively (Figure 7e). These findings suggest that local calibration or at least a local calibration audit is necessary before using absolute‐risk estimates to support site‐specific review or triage decisions. The 300‐case Platt calibration analysis should be interpreted as an implementation demonstration rather than a minimum deployment requirement; smaller calibration‐in‐the‐large audits, regional pooled calibration, quality‐aware recalibration and uncertainty routing may be more feasible in resource‐limited workflows (Table S17).

Risk quartiles separated the observed progression. In UKB internal testing, 5‐year incidence increased from 0.38% in the lowest quartile to 7.20% in the highest quartile. In TMUEH external testing, the corresponding increase was from 0.20% to 6.71% (Figure 5c and Table S11). At a 5% UKB operating threshold, 1394 of 11 553 baseline non‐late participants were referred for enhanced review, corresponding to 12.1% workload, sensitivity 56.4%, specificity 89.0%, and PPV 11.4% (Figure 5f and Table S6). At a 6% TMUEH threshold, workload was 8.7%, sensitivity 30.8%, specificity 91.9%, and PPV 9.4%. The TMUEH 6% threshold therefore represents a lower‐workload external operating point rather than a high‐sensitivity screening threshold. Decision‐curve analysis showed positive net benefit across a clinically plausible threshold range, but the absolute magnitude of net benefit remained modest in the external cohort because of low event prevalence and limited progression‐event counts (Figure 5d,e).

### 2.6 Interpretability, Lesion‐Informed Risk Patterns and Error Phenotypes

Permutation analysis ranked drusen volume, drusen area, ellipsoid‐zone disruption, age/systemic‐risk context, and hyperreflective foci as the most influential features for progression prediction (Figure 6a). Partial‐dependence curves showed nonlinear increases in predicted 5‐year risk with higher drusen volume and greater tissue disruption (Figure 6b,c). These patterns support a lesion‐informed interpretation of the model: drusen burden represents the early/intermediate disease substrate, pigment abnormality and hyperreflective foci indicate retinal stress, ellipsoid‐zone disruption reflects outer‐retinal vulnerability, and GA or exudative fluid indicates late‐stage lesions. These concept‐level outputs should be interpreted as structured predictive explanations rather than causal evidence of disease mechanisms.

**FIGURE 6:**
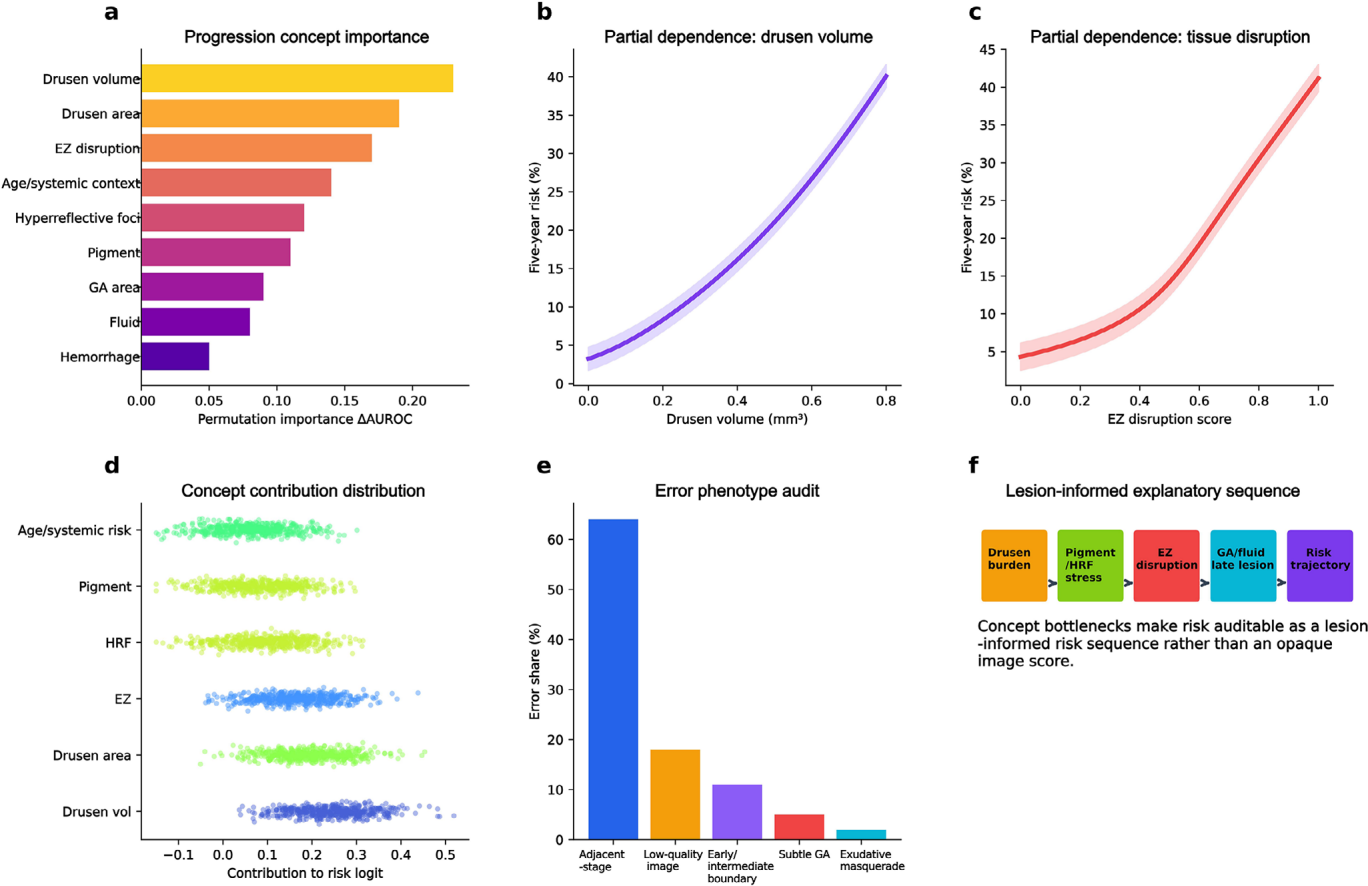
Interpretability and Lesion‐informed Risk Patterns. (a) Permutation Importance of Lesion‐related and Clinical‐context Concepts for Five‐year Progression Prediction. (b, c) Partial‐dependence Curves Showing the Relationship of Drusen Volume and Ellipsoid‐zone Disruption With Predicted Five‐year Risk. (d) Distribution of Concept Contributions to the Risk Logit. (e) Error Phenotype Audit Showing the Proportion of Errors Associated With Adjacent‐stage Disagreement, Low‐quality Imaging, Early/Intermediate Boundary Cases, Subtle Geographic Atrophy and Exudative Masquerade. (f) Lesion‐informed Explanatory Sequence Linking Drusen Burden, Pigment/HRF Stress, EZ Disruption, Late Lesions and Risk Trajectory. This Schematic Is Intended as an Explanatory Model of Prediction Behavior Rather Than Proof of Causal Disease Mechanisms. AMD, Age‐related Macular Degeneration; HRF, Hyperreflective Foci; EZ, Ellipsoid Zone; GA, Geographic Atrophy.

Concept contribution distributions were heterogeneous across participants (Figure 6d). Some high‐risk individuals were primarily driven by drusen volume, whereas others were driven by ellipsoid‐zone disruption or age/systemic‐risk context. This heterogeneity is clinically relevant because it suggests that ORBIT‐AMD can support different review pathways, including routine monitoring for low‐burden low‐risk eyes, closer OCT surveillance for high drusen or tissue‐disruption eyes, and urgent retina review for suspected late lesions.

Error phenotyping showed that 64% of errors were adjacent‐stage disagreements, 18% were associated with low‐quality imaging, 11% were early/intermediate boundary cases, 5% were subtle GA, and 2% were exudative masquerades (Figure 6e). These errors are consistent with clinically meaningful boundary ambiguity rather than random failure. The schematic interpretation in Figure 6f should therefore be viewed as a lesion‐informed explanatory sequence for model behavior rather than proof of causal disease mechanisms.

### 2.7 Protocol Alignment, Ablation and Robustness

Counterfactual protocol alignment reduced cohort separability while retaining disease‐discrimination signals. Cohort maximum mean discrepancy decreased from 0.42 in raw style‐sensitive representations to 0.21 after masked encoding and 0.06 after alignment (Figure 7a). The adversarial cohort critic approached chance‐level accuracy by late training (Figure 7b), and the aligned latent projection showed greater overlap between UKB and TMUEH samples (Figure 7c). Distributional‐shift auditing showed reduced standardized distances for age, image quality, stage, drusen burden, and follow‐up after alignment (Figure 7d and Table S16). Because age and disease biology are partly entangled with cohort source, this result should be interpreted as reduced cohort separability rather than proof that only acquisition artifacts were removed. Age‐stratified and quality‐stratified performance remained close to the overall external estimate, supporting partial preservation of clinically relevant signal after alignment (Figure 8b, Figure S6, and Table S7).

**FIGURE 7:**
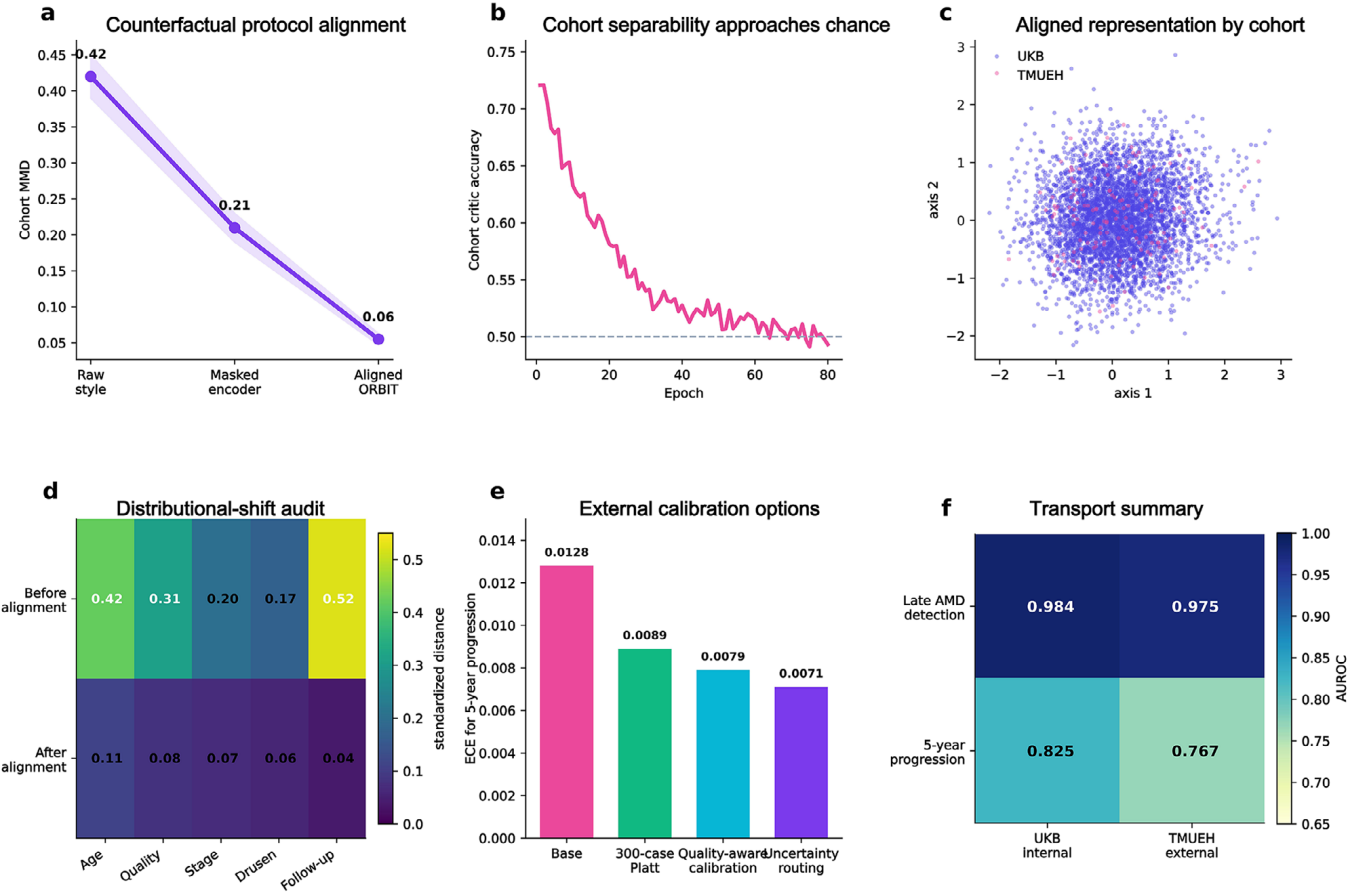
Protocol Alignment Under Cohort Shift. (a) Reduction in cohort maximum mean discrepancy across raw style‐sensitive representations, masked encoder representations, and aligned ORBIT‐AMD representations. (b) Cohort‐critic accuracy approaching chance level during adversarial alignment. (c) Aligned latent representation by cohort. (d) Distributional‐shift audit before and after alignment. (e) External calibration options for 5‐year progression prediction. (f) Transport summary for prevalent late‐AMD detection and 5‐year progression prediction across UKB internal and TMUEH external testing. These analyses evaluate reduced cohort separability and external robustness under the present cohort shift but do not establish universal scanner, site, or population invariance. Because age, disease severity, and protocol source may be correlated across cohorts, reduced standardized age distance should be interpreted as part of the cohort‐shift audit rather than proof that biological age information was removed or preserved perfectly. MMD, maximum mean discrepancy; ECE, expected calibration error; AUROC, area under the receiver operating characteristic curve; AMD, age‐related macular degeneration.

**FIGURE 8:**
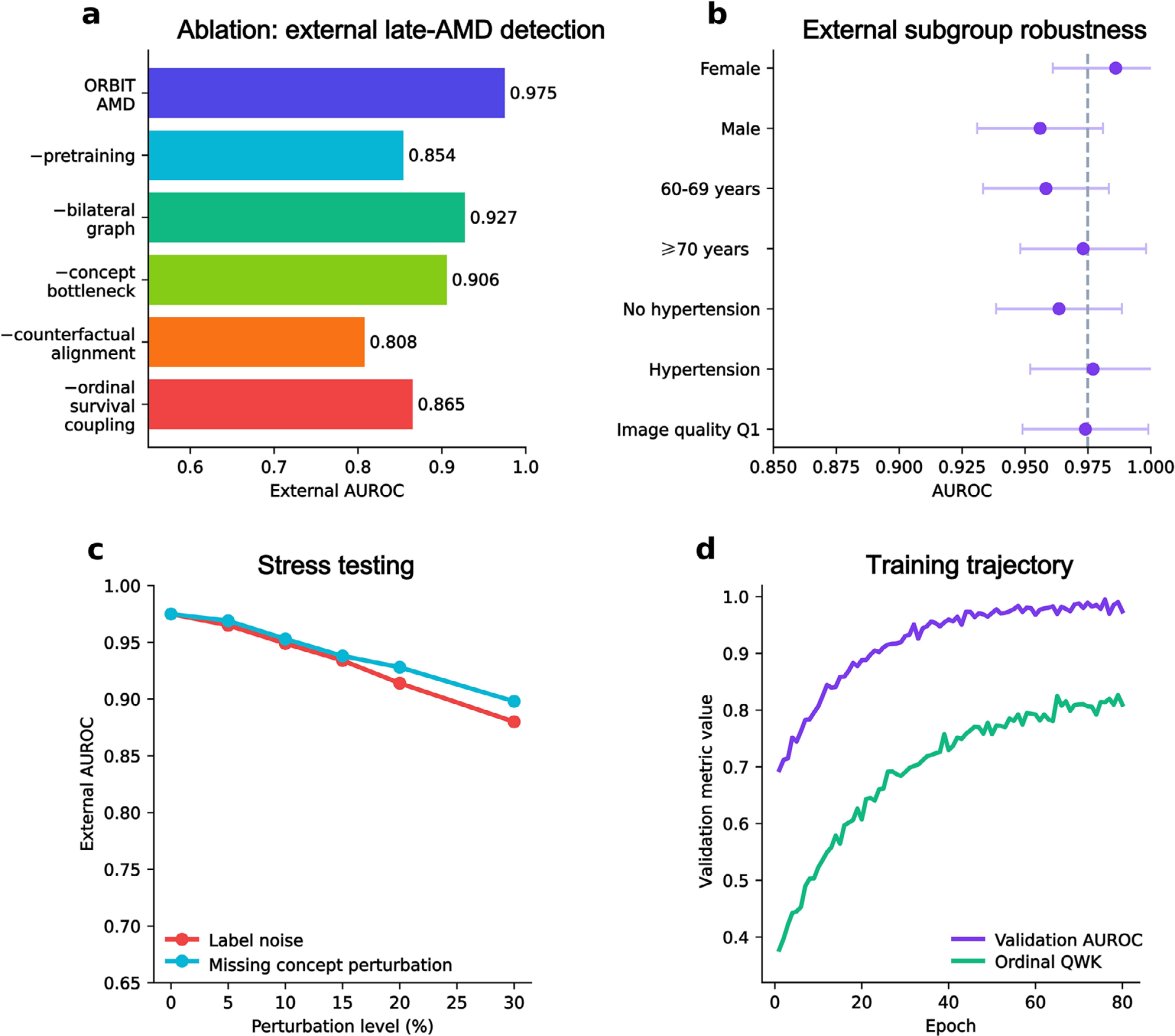
Component Ablations, Robustness, and Calibration Diagnostics. (a) External late‐AMD detection AUROC after removing individual ORBIT‐AMD components. (b) External subgroup AUROC estimates with confidence intervals. (c) Stress testing under label noise and missing‐concept perturbation. (d) Training trajectory for validation AUROC and ordinal quadratic weighted kappa, shown as validation metric values across training epochs. AUROC, area under the receiver operating characteristic curve; QWK, quadratic weighted kappa; AMD, age‐related macular degeneration.

External performance remained high for prevalent late‐AMD detection but was more attenuated for progression prediction. The external AUROC was 0.975 for late‐AMD detection and 0.767 for 5‐year progression (Figure 7f). Subgroup analysis showed AUROC values near the overall external estimate for women, participants aged at least 70 years, hypertensive participants, and the lowest image‐quality quartile (Figure 8b, Figure S6, and Table S7). However, several subgroups contained few late‐AMD or progression events, and these estimates should therefore be interpreted as exploratory rather than definitive subgroup evidence.

Component ablations supported the contribution of the full model design (Figure 8a, Figure S7, and Table S8). Removing masked pretraining reduced external late‐detection AUROC from 0.975 to 0.854. Removing bilateral graph attention reduced AUROC to 0.927, removing the concept bottleneck to 0.906, removing counterfactual alignment to 0.808, and removing ordinal‐survival coupling to 0.865. These results suggest that performance depended on the combination of foundation representation, bilateral context, interpretable concepts, trajectory coupling, and protocol alignment. Stress tests under label noise and concept perturbation showed gradual degradation rather than abrupt collapse (Figure 8 and Figures S8 and S9), although these analyses should be interpreted as robustness diagnostics rather than evidence of prospective deployment readiness. Confidence intervals are reported for primary ORBIT‐AMD metrics, while baseline and ablation models are presented as exploratory component‐diagnostic point estimates when resampled prediction outputs are not available for all variants (Tables S5 and S8).

## 3 Discussion

This study reframes AMD machine learning as trajectory learning rather than static image classification. The main contribution of ORBIT‐AMD is that it links prevalent disease stage, bilateral asymmetry, lesion‐level concepts, future progression, calibration, and threshold‐dependent workload in a single auditable framework. This structure is clinically relevant because AMD is managed as a sequence of risk states. A binary classifier may identify obvious late disease, but it cannot by itself define surveillance intervals, communicate progression risk, or explain whether risk is driven by drusen burden, tissue disruption, atrophy, or exudation. The TMUEH external‐validation cohort strengthens the clinical relevance of the study because it reflects an older screening and routine‐clinic population rather than a disease‐enriched case‐control test set. Any AMD accounted for 14.0% and late AMD for 1.45% of this cohort. This setting makes precision‐recall, PPV, and workload analyses more informative than AUROC alone. At the same time, it makes external validation more difficult because the number of late‐AMD and progression events is limited, widening uncertainty intervals and stressing calibration.

The reduction in progression AUROC from 0.825 in the UKB internal test set to 0.767 in the TMUEH external test set should be interpreted in the context of simultaneous cohort differences rather than as a simple internal‐to‐external performance gap. The external cohort differed in age, clinical setting, follow‐up structure, endpoint source, imaging workflow, and event scarcity. These factors are particularly influential for longitudinal progression prediction, because future late‐AMD conversion depends not only on baseline retinal appearance but also on surveillance intensity, treatment exposure, censoring, and competing health events.

The model's interpretability results are consistent with established lesion‐based understanding of AMD progression, but they should not be interpreted as proof of causal mechanisms. Drusen burden and pigment abnormality form the early/intermediate disease substrate, hyperreflective foci and ellipsoid‐zone disruption indicate retinal stress and outer‐retinal vulnerability, and GA or exudative fluid defines late endpoints [25, 26, 27]. The model's errors were mainly adjacent‐stage or boundary errors, which is biologically plausible because AMD stages are discrete clinical labels imposed on a continuous disease process. The separation of progression risk across quartiles further suggests that the learned trajectory contains prognostic information even when exact ordinal staging is imperfect. Recent bioinformatics studies have also highlighted immune‐metabolic and epigenetic regulatory processes, including lactylation‐related signatures, as potential contributors to AMD pathogenesis [28]. These findings support the broader biological relevance of interpretable lesion‐ and biology‐informed risk models, while also emphasizing that predictive retinal‐imaging models do not by themselves establish molecular causality.

The concept bottleneck is an important design feature because it embeds interpretability into the predictive architecture rather than relying only on posthoc visualization [29]. Saliency maps can indicate image regions that influence a prediction, but they do not necessarily identify which clinical lesion mediated the decision [30]. ORBIT‐AMD exposes intermediate lesion‐related variables, allowing investigators to distinguish drusen‐driven risk, tissue‐disruption risk, late‐lesion risk, and uncertainty related to low‐quality scans. This distinction is particularly relevant in AMD because different lesions imply different biological processes, follow‐up needs, and clinical actions.

Exact recognition of early and intermediate AMD remains the main classification weakness. This limitation is clinically important because early/intermediate AMD often determines surveillance intervals, lifestyle counselling, and trial eligibility. However, these categories are also the most label‐sensitive stages because drusen size, pigmentary abnormality, hyperreflective foci, and subtle outer‐retinal changes exist on a continuum. The relatively low class‐specific recall for early and intermediate AMD therefore suggests that ORBIT‐AMD should be used to prioritize review and quantify risk rather than to replace specialist stage assignment. Future work could improve minority‐stage recall using enriched intermediate‐AMD training samples, class‐balanced ordinal losses, uncertainty‐triggered human review, and reading‐center adjudication of borderline cases.

The bilateral graph is also clinically meaningful. Fellow‐eye status is among the most important sources of AMD prognostic information [31]. By modeling both eyes as a graph with learned attention, ORBIT‐AMD can represent asymmetry, fellow‐eye conversion, and inter‐eye risk propagation. This improves over approaches that average eyes, select one eye, or treat each eye independently. The benefit is not merely higher discrimination; it is a closer match to retina‐specialist reasoning.

Protocol alignment is another important component of the framework. Ophthalmic AI models may learn acquisition‐specific shortcuts when images are collected using different cameras, OCT devices, or clinical workflows [32, 33]. ORBIT‐AMD used device and protocol information for eligibility, audit, and stress testing, but withheld device identity from prediction heads. Counterfactual alignment penalized representations that separated cohorts primarily by acquisition style [34]. These results indicate reduced cohort separability and improved external robustness, but they do not prove universal scanner or site invariance. Importantly, acquisition protocol, age, and disease severity may be partially correlated across real‐world cohorts. Therefore, the observed reduction in age‐related standardized distance after alignment should be interpreted cautiously: it indicates reduced cohort separability, not definitive proof that only nuisance style information was removed. The preserved age‐stratified and quality‐stratified performance suggests that a clinically relevant signal was at least partly retained, but prospective multi‐device validation remains necessary.

Clinically, ORBIT‐AMD should be interpreted as a decision‐support framework rather than an autonomous diagnostic system. In population‐imaging settings, it could prioritize eyes for expert review, trigger OCT confirmation, personalize follow‐up intervals or enrich prevention trials. In hospital‐based workflows, it could help separate low‐risk drusen from higher‐risk intermediate disease and identify uncertain scans requiring specialist review. The decision‐curve and threshold analyses connect risk scores with workload, sensitivity, specificity, and PPV, showing that the same AUROC can have different clinical consequences depending on prevalence, calibration, and operating threshold.

The local recalibration analysis also has practical deployment implications. The 300‐case Platt calibration experiment was designed to demonstrate the value of site‐specific recalibration rather than to define a universal minimum sample size. In resource‐limited settings, a more realistic implementation pathway may begin with calibration‐in‐the‐large using a smaller local audit set, periodic drift monitoring, uncertainty routing for ambiguous cases, and regional pooled recalibration across similar clinics. When adequate longitudinal outcome data are not yet available, the model should be used primarily for relative risk ranking and review prioritization rather than for communicating precise absolute 5‐year risk. Because the study was designed for prediction rather than causal inference, age, image quality, systemic comorbidity, and cohort source were evaluated primarily as sources of transportability, calibration, and subgroup robustness rather than as confounders requiring causal adjustment. This distinction is important when interpreting the subgroup, alignment, and calibration analyses: they assess whether model performance is stable across clinically relevant strata, but they do not estimate causal effects of these factors on AMD progression. Taken together, these results reinforce that 5‐year progression prediction should be interpreted as a transport‐sensitive prognostic task requiring local calibration audit, drift monitoring, and prospective workflow evaluation before deployment.

## 4 Study Limitations

This study has several limitations. First, UKB and TMUEH differed in study setting, age distribution, disease prevalence, imaging workflow, follow‐up structure, and endpoint ascertainment. These differences strengthen external validation but also introduce residual dataset shift that cannot be fully removed by protocol alignment.

Second, endpoint and concept labels were harmonized but not identically generated across cohorts. UKB lesion concepts were algorithmic image‐derived estimates from a fixed concept‐extraction workflow rather than exhaustive manual reading‐center annotations, whereas TMUEH endpoints were supported by clinical diagnosis, CFP/OCT review, follow‐up imaging, and treatment records. This label‐source heterogeneity may contribute to the lower external performance for 5‐year progression prediction and limit the interpretation of concept outputs as definitive lesion measurements.

Third, the TMUEH cohort included only 29 baseline late‐AMD cases and 52 five‐year progression events. This limited the precision of external AUROC, AUPRC, calibration, subgroup, and threshold‐specific estimates. The high external AUROC for prevalent late AMD should therefore be interpreted together with precision‐recall behavior, PPV, confidence intervals, and missed‐case review.

Fourth, exact four‐stage ordinal classification was weakest for early and intermediate AMD, probably reflecting both the biological continuity of AMD progression and boundary uncertainty around drusen burden, pigmentary abnormality, and subtle OCT lesions. ORBIT‐AMD should therefore be used for risk stratification and review prioritization rather than as a replacement for specialist staging, especially for early/intermediate boundary cases.

Fifth, the 5‐year progression prediction used a cause‐specific discrete‐time survival formulation. Death, loss to follow‐up, and administrative end of follow‐up were treated as censoring, and death was not explicitly modeled as a competing risk. Harmonized mortality‐specific censoring counts were not available across both cohorts, so we could not quantify the effect of competing mortality on absolute 5‐year risk. This limitation may lead to overestimation of cumulative progression probabilities, particularly in older or more comorbid external populations. Future prospective validation should include mortality, treatment exposure, and other competing‐event information when available.

Sixth, protocol alignment reduced cohort separability but cannot prove complete disentanglement of acquisition style from biological cohort differences such as age or disease severity. Concept bottlenecks improve auditability but remain dependent on image quality, segmentation reliability, modality availability, and the concept‐extraction pipeline. These outputs should be viewed as structured explanatory estimates, not causal mechanisms or substitutes for specialist grading.

In addition, although baseline and ablation‐model comparisons supported the contribution of the full ORBIT‐AMD architecture, 95% confidence intervals were not available for all baseline and ablated variants. Therefore, the statistical significance of ORBIT‐AMD's apparent performance superiority over these ablated variants could not be formally established. These comparisons should be interpreted as exploratory component‐diagnostic point estimates rather than statistically confirmed evidence of superiority.

Finally, 5‐year progression prediction may also be affected by shorter TMUEH follow‐up, differential surveillance, anti‐VEGF treatment exposure, cataract or media opacity, refractive status, prior retinal interventions, and incomplete treatment capture. Local recalibration was demonstrated in the external cohort, but implementation in resource‐limited settings may require smaller calibration audits, pooled regional calibration, uncertainty routing, or prospective drift monitoring. ORBIT‐AMD should be considered a decision‐support tool requiring prospective validation, local calibration assessment, and workflow evaluation before deployment.

## 5 Conclusions

ORBIT‐AMD is a multi‐cohort AMD machine‐learning framework that combines masked retinal representation learning, bilateral eye‐graph modeling, concept bottlenecks, ordinal staging, cause‐specific discrete‐time survival prediction, and counterfactual protocol alignment. In real‐world UKB and TMUEH retinal cohorts, ORBIT‐AMD showed strong discrimination for prevalent late‐AMD detection and moderate external performance for 5‐year progression prediction, while providing auditable lesion‐level explanations, calibration assessment and threshold‐dependent workload estimates. These results support trajectory‐aware multimodal learning as a clinically structured approach for AMD risk stratification and review prioritization. Prospective validation, local calibration assessment, competing‐risk‐aware evaluation and workflow testing are required before clinical deployment.

## 6 Methods

### 6.1 Study Design

We conducted a real‐world, dual‐cohort prediction‐model development and external‐validation study. UKB was used for model development, validation, and internal testing, and TMUEH was used as an independent external validation cohort. The primary endpoint was prevalent late‐AMD detection at baseline. Secondary endpoints were four‐stage ordinal AMD classification, 5‐year progression from baseline nonlate AMD to late AMD, calibration, risk‐quartile separation, decision‐curve net benefit, workload at selected operating thresholds, subgroup robustness, and component ablation.

The analysis followed a participant‐level design. Both eyes were considered when eligible, but all data splitting, outcome assignment, and performance evaluation were performed at the participant level to prevent inter‐eye information leakage. UKB was divided into training, validation, and internal‐test sets in a 70/10/20 ratio using stage‐aware participant‐level stratification, with baseline AMD stage and incident late‐AMD status among baseline non‐late participants checked across splits (Table S13). No TMUEH data were used for model training, feature selection, hyperparameter tuning, or threshold selection. When local recalibration was evaluated, it was reported as an exploratory deployment analysis because recalibration and external evaluation were performed within the same external cohort (Tables S10 and S17).

Because this was a retrospective real‐world prediction‐model study using all eligible participants with protocol‐compatible imaging and follow‐up, no conventional trial‐style power calculation was performed. The effective sample size was determined by available UKB and TMUEH imaging, stage labels, and longitudinal endpoints after predefined exclusions. Precision was therefore reported using participant‐level bootstrap confidence intervals for primary ORBIT‐AMD metrics and by reporting event counts for discrimination, calibration, subgroup, and threshold analyses. No intervention, treatment allocation, or trial‐style randomization was performed because this was a retrospective prediction‐model development and validation study. Blinding was therefore not applicable in the interventional sense; however, all model development, threshold selection, and hyperparameter tuning were performed without using the TMUEH external‐validation labels.

### 6.2 Cohort Sources, Eligibility and Participant Flow

The UKB cohort was derived from the UK Biobank baseline retinal imaging assessment, a standardized population‐based ophthalmic imaging resource linked to baseline questionnaire data, physical measurements, and longitudinal health‐record data. The baseline date was defined as the date of retinal imaging. Retinal imaging in UKB included color fundus photography (CFP) and spectral‐domain optical coherence tomography (OCT) acquired under the UKB ophthalmic imaging protocol. CFP and OCT images were used jointly for AMD stage assignment, lesion‐concept estimation, and model input construction. Demographic and systemic covariates were obtained from baseline assessment data and linked health records.

Participants were eligible for the UKB analysis if they had at least one gradable eye with sufficient retinal imaging information for AMD stage assignment. Participants were excluded if they had missing core imaging information, ungradable CFP or OCT images, images outside the predefined imaging protocol, non‐AMD macular comorbidity that could confound AMD classification, missing key demographic or clinical covariates, or insufficient follow‐up information for progression analysis. After exclusions, the UKB analysis cohort included 58 214 participants and 109 691 eligible eyes. Participants were split at the participant level into training, validation, and internal‐test sets, yielding 40 750, 5821, and 11 643 participants, respectively.

The TMUEH cohort was an independent hospital‐based external‐validation cohort derived from retinal imaging performed in screening and routine‐clinic workflows at Tianjin Medical University Eye Hospital. The source population comprised eligible retinal imaging episodes recorded between January 2018 and December 2024. CFP and OCT were acquired using routine institutional imaging devices and protocols. If more than one imaging device or acquisition protocol was used, device and protocol information was retained for eligibility assessment, quality control, sensitivity analysis, and drift audit, but was not used as a direct prediction feature.

The baseline date for TMUEH participants was defined as the first eligible retinal‐imaging visit during the study period. Repeat non‐baseline visits were not treated as independent baseline samples. Instead, follow‐up imaging, clinical diagnoses, and treatment records were used to ascertain incident late AMD when available. Participants were eligible if they had gradable CFP and OCT images and sufficient clinical information for AMD stage assignment. Participants were excluded if they had ungradable imaging, incomplete baseline imaging, images obtained outside the locked workflow, alternative macular diagnoses that could confound AMD classification, or insufficient information for endpoint adjudication. The final TMUEH cohort included 1996 participants and 3780 eligible eyes and was used only for external validation.

### 6.3 AMD Diagnostic Criteria and Endpoints

AMD stage was assigned at the participant level using the worse eye. Four ordinal stages were defined: no AMD, early AMD, intermediate AMD, and late AMD. Early AMD was characterized by small‐to‐medium drusen or mild pigmentary abnormality without late lesions. Intermediate AMD was defined by larger drusen burden, pigment abnormality, hyperreflective foci, ellipsoid‐zone disturbance, or subretinal drusenoid deposits without GA or nAMD. Late AMD was defined by GA or nAMD‐compatible exudative features, including intraretinal fluid, subretinal fluid, subretinal hyperreflective material, hemorrhage, fibrovascular pigment epithelial detachment, or other exudative macular features consistent with neovascular disease.

The prevalent late‐AMD endpoint was defined as late AMD at baseline and was the primary prediction endpoint. The longitudinal endpoint was incident late AMD within five years among participants without late AMD at baseline and was treated as a secondary prognostic endpoint. Time at risk began at baseline retinal imaging and ended at the first documented late‐AMD event, last available retinal follow‐up, death, loss to follow‐up, or administrative censoring, whichever occurred first. The survival head estimated cause‐specific discrete‐time risk under independent censoring; death was not modeled as a competing event in the primary architecture (Table S14). Participants with late AMD at baseline were excluded from progression analyses but retained for prevalent late‐AMD detection and ordinal AMD staging analyses.

Endpoint ascertainment differed by cohort because of the nature of the data sources. In UKB, baseline AMD stage and late‐AMD events were derived from harmonized retinal image‐derived information and linked diagnostic records where available. In TMUEH, AMD stage and late‐AMD events were supported by clinical CFP/OCT review, routine ophthalmic diagnosis, and institutional follow‐up records. To improve cross‐cohort comparability, the same four‐level AMD staging framework and the same participant‐level worse‐eye rule were applied in both cohorts.

### 6.4 Image Grading and Endpoint Adjudication

Baseline AMD stage was assigned at the participant level using a harmonized worse‐eye rule. Each eligible eye was first assigned an eye‐level AMD stage based on available CFP/OCT‐derived information and clinical records where applicable. The participant‐level stage was then defined by the more severe eye. For participants with only one eligible eye, the available eye determined the participant‐level stage.

The same four‐level AMD staging framework was applied in both cohorts. No AMD was defined as the absence of AMD‐compatible drusen, pigmentary abnormality, or late lesions. Early AMD was defined by small‐to‐medium drusen or mild pigmentary abnormality without late lesions. Intermediate AMD was defined by larger drusen burden, pigmentary abnormality, hyperreflective foci, ellipsoid‐zone disturbance, or subretinal drusenoid deposits without geographic atrophy (GA) or neovascular AMD (nAMD). Late AMD was defined by GA or nAMD‐compatible exudative lesions.

GA was identified by AMD‐compatible atrophic macular change, including well‐demarcated retinal pigment epithelium and outer‐retinal atrophy on CFP/OCT, without evidence of active neovascular exudation. nAMD was identified by exudative macular features, including intraretinal fluid, subretinal fluid, subretinal hyperreflective material, hemorrhage, fibrovascular pigment epithelial detachment, macular neovascularization recorded in clinical diagnosis, or treatment evidence compatible with neovascular disease. For subtype summaries, participants with definite neovascular features were classified as nAMD; otherwise, late atrophic lesions were classified as GA. Both GA and nAMD were included under the broader late‐AMD endpoint.

Endpoint ascertainment differed by cohort because of the underlying data structure. In UKB, baseline AMD stage and late‐AMD events were derived from a harmonized combination of retinal image‐derived information and linked diagnostic records where available. Image‐derived evidence was prioritized for baseline stage assignment because baseline retinal imaging directly captured disease status at the imaging visit. Linked diagnostic records were used to support longitudinal late‐AMD event ascertainment. When both image‐derived and linked‐record evidence were available, the earliest credible date indicating late AMD was used as the event date. Ambiguous cases with alternative macular diagnoses or insufficient evidence for AMD subtype assignment were excluded or retained only in sensitivity analyses according to predefined eligibility rules.

In TMUEH, AMD stage and late‐AMD endpoints were ascertained using the institutional clinical standard based on retinal‐specialist diagnosis, same‐visit CFP/OCT review, follow‐up imaging, diagnostic records, and treatment information where available. Baseline CFP and OCT were reviewed within routine specialist clinical care, and the baseline AMD stage was assigned using the same four‐level staging framework and participant‐level worse‐eye rule as in UKB. Follow‐up visits were used to identify incident late AMD, with late‐AMD conversion defined as the first documented evidence of GA or nAMD on follow‐up imaging or clinical records. For uncertain cases, endpoint assignment required concordant clinical evidence from retinal imaging, specialist diagnosis, or treatment‐related records. Cases with insufficient evidence to distinguish AMD from alternative macular disease, or with unclear GA/nAMD subtype assignment, were excluded from the primary endpoint analysis or retained only in sensitivity analyses according to predefined eligibility rules. Thus, TMUEH endpoints were based on real‐world clinical adjudication within routine ophthalmic practice rather than retrospective manual grading of every lesion by a centralized reading center.

For progression analyses, the event date was defined as the first date on which late AMD was documented by follow‐up imaging, clinical diagnosis, treatment record, or linked diagnostic record. Participants without late AMD were censored at their last available retinal follow‐up, death, loss to follow‐up, or administrative censoring, whichever occurred first. Participants censored before five years without late AMD contributed follow‐up information up to the censoring time and were not treated as confirmed five‐year non‐progressors. To make label provenance explicit, each cohort was mapped according to baseline stage source, late‐AMD subtype source, progression‐date source, concept‐label source, and expected bias direction (Table S12). UKB labels were derived from protocolized image‐derived information and linked diagnostic records where available; TMUEH labels were derived from retinal‐specialist clinical diagnosis, same‐visit CFP/OCT review, follow‐up imaging, and treatment records. This harmonisation allowed a common four‐stage endpoint but did not eliminate label‐source heterogeneity.

### 6.5 Concept Source, Extraction, and Validation

The concept bottleneck was designed to represent clinically meaningful AMD lesion patterns visible on CFP and OCT rather than to provide definitive manual lesion measurements. The concept set included drusen area, drusen volume, pigment disturbance, hyperreflective foci, ellipsoid‐zone disruption, GA area, exudative fluid volume, and hemorrhage. Continuous concepts, including drusen area, drusen volume, GA area, and fluid volume, were represented as standardized quantitative estimates. Binary or semi‐quantitative concepts, including pigment disturbance, hyperreflective foci, ellipsoid‐zone disruption, and hemorrhage, were represented as presence/absence or normalized severity indicators.

Concept variables were generated using a predefined image‐processing and model‐based concept‐extraction workflow applied to CFP and OCT data. This workflow was implemented as an internal, fixed algorithmic pipeline rather than as a manual reading‐center grading system. For CFP, the pipeline used macular‐field cropping, vessel/optic‐disc masking where needed, intensity normalization, and lesion‐oriented segmentation or scoring to estimate drusen area, pigment disturbance, and hemorrhage. For OCT, volumetric B‐scan processing was used to estimate drusen volume, hyperreflective foci burden, ellipsoid‐zone disruption, GA‐compatible atrophic change, and exudative fluid volume. Continuous concepts were standardized within the UKB training set and applied unchanged to validation, internal‐test, and TMUEH external‐test data; binary or semi‐quantitative concepts were coded in the same direction, with larger values indicating greater lesion burden or disruption. The concept‐extraction workflow was developed and fixed before downstream outcome modeling. It was trained and tuned using the UKB training and validation data only, together with image‐derived lesion information available within the development cohort. No TMUEH outcome labels, AMD stage labels, or progression endpoints were used to train, tune, or select the concept‐extraction workflow. The TMUEH cohort was therefore reserved for external validation and external clinical plausibility audit.

Because UKB is a large‐scale population imaging cohort, exhaustive manual adjudication of every lesion concept was not available for all participants. Therefore, UKB lesion concepts were treated as image‐derived algorithmic estimates rather than definitive manual annotations. These estimates were used to support concept bottleneck learning, stage‐conditioned lesion‐gradient analysis, and model interpretability. In TMUEH, routine clinical CFP/OCT review, retinal‐specialist diagnosis, follow‐up imaging, and treatment records provided external clinical context for auditing whether the concept estimates followed plausible AMD lesion patterns.

Concept validity was evaluated in three complementary ways. First, stage‐conditioned concept gradients were examined to determine whether drusen burden, pigment disturbance, hyperreflective foci, ellipsoid‐zone disruption, GA, and exudative fluid followed clinically plausible severity patterns across no AMD, early AMD, intermediate AMD, and late AMD. Second, concept‐label correlations were calculated to assess alignment between concept estimates and AMD stage or lesion information where available. Third, concept contribution distributions and error phenotypes were examined to determine whether model predictions were driven by clinically interpretable lesion pathways. The concept bottleneck was therefore interpreted as an auditable explanation layer, not as proof of causal disease mechanisms or as a replacement for specialist image grading.

### 6.6 Retinal Concepts and Feature Construction

Each eligible eye contributed CFP‐ and OCT‐derived concepts. The concept set included drusen area, drusen volume, pigment disturbance, hyperreflective foci, ellipsoid‐zone disruption, GA area, exudative fluid volume, and hemorrhage. Continuous concepts were represented as standardized quantitative measures, and binary concepts were represented as presence or absence indicators. Participant‐level summaries incorporated both the worse‐eye burden and inter‐eye asymmetry.

For bilateral modeling, each participant was represented as a two‐node graph whenever both eyes were eligible. If only one eye was eligible, the missing eye node was masked. Let *Zi,L* and *Zi,R* denote left‐ and right‐eye embeddings. The participant representation was computed using attention‐weighted bilateral aggregation:

$$\;h_{i}=\sum_{e\in \left\{L,R\right\}}a_{i,e}\;z_{i,e},\;\;\;a_{i,e}=\frac{exp\left(q^{⊤}z_{i,e}\right)}{\sum_{e^{\prime }\in \left\{L,R\right\}}exp\left(q^{⊤}z_{i,e^{\prime }}\right)}\;$$

where *a_i,e_* is the learned attention weight for eye *e*, and *q* is the attention query vector. This allowed the model to emphasize the more informative or more advanced eye while retaining fellow‐eye context.

### 6.7 ORBIT‐AMD Architecture

ORBIT‐AMD was specified as a multimodal retinal learning framework using CFP and OCT under harmonized acquisition and quality‐control rules. CFP images were resized to 512 × 512 pixels. OCT volumes were represented by 64 B‐scans resized to 224 × 224 pixels. Image intensities were normalized within modality. Images failing predefined quality‐control criteria were excluded before model training or masked when only one eye was eligible.

During training, image augmentation was applied to the training set only, including random cropping, horizontal flipping where anatomically appropriate, brightness and contrast perturbation, and mild geometric transformation. No augmentation was applied to validation, internal‐test, or external‐test images.

The encoder used 16 × 16 image patches, 12 transformer blocks, hidden dimension 768, 12 attention heads and dropout 0.10. CFP and OCT representations were fused after modality‐specific encoding, followed by bilateral graph aggregation and concept bottleneck prediction. The model produced three participant‐level outputs: prevalent late‐AMD probability, four‐stage ordinal AMD classification and discrete‐time progression risk.

### 6.8 Masked Representation Learning

Masked representation learning was used to initialize the retinal encoder. The CFP masking ratio was 65%, and the OCT token masking ratio was 50%. Reconstruction loss was computed only over masked tokens:

$$\{L_{\mathrm{rec}}=\frac{1}{\left|M\right|}\;\sum_{m\in M}{\left\|x_{m}-{\hat{x}}_{m}\right\|}_{2}^{2}$$

where *M* denotes the set of masked tokens, *Xm* is the original token and *X^m* is the reconstructed token. The pretrained encoder was then fine‐tuned for concept prediction, ordinal staging, and progression prediction.

### 6.9 Concept Bottleneck Learning

The concept bottleneck predicted AMD lesion‐concept estimates from the participant representation. Continuous concept estimates were trained using mean‐squared error, whereas binary or semi‐quantitative concept estimates were trained using binary cross‐entropy or ordinal‐compatible loss functions as appropriate. The concept bottleneck served two purposes. First, it provided intermediate lesion‐level features for downstream ordinal staging and survival prediction. Second, it allowed model outputs to be interpreted through clinically recognizable AMD lesion pathways.

Concept outputs were treated as structured explanatory estimates rather than definitive clinical measurements. Downstream interpretation therefore focused on whether predicted risk was mediated through clinically plausible concept patterns, such as drusen burden, pigment disturbance, hyperreflective foci, ellipsoid‐zone disruption, GA, or exudative fluid, rather than assuming that the concept bottleneck provided manual‐grade lesion measurements.

### 6.10 Ordinal AMD Staging

AMD stage was modeled as an ordered outcome rather than a nominal class. ORBIT‐AMD used a cumulative‐logit ordinal head:

$$\mathrm{Pr}\left(Y_{i}\leq k|h_{i}\right)=\sigma \left(\theta_{k}-\beta^{⊤}h_{i}\right),\;\;k=0,1,2,$$

where *Yi* is the four‐level AMD stage, *θk* are ordered thresholds, *β* is the stage‐risk direction, and *σ(·)* is the logistic function. This formulation enforces an ordered severity structure and reduces biologically implausible no‐AMD to late‐AMD jumps.

### 6.11 Discrete‐Time Survival Prediction

For baseline non‐late participants, five‐year progression risk was modeled using discrete‐time hazards. Follow‐up was divided into six intervals: 0–1, 1–2, 2–3, 3–5, 5–7, and 7–10 years. Participants who died, were lost to follow‐up, or reached administrative censoring before developing late AMD contributed risk‐set information until the censoring interval. Death was not modeled as a competing event in the primary architecture (Table S14). The interval‐specific hazard was defined as:

$$\;\lambda_{i,t}=\mathrm{Pr}\left(\;T_{i}\in I_{t}|T_{i}\geq t,h_{i}\right)=\sigma \left(\gamma_{t}+w^{⊤}h_{i}\right),$$

where *I_t_* is the time interval, *γt* is the baseline logit hazard for interval *t*, and *w^⊤^hi* is the participant‐specific risk score.

The cumulative risk by time τ was calculated as:

$\mathrm{Pr}\left(T_{i}\leq \tau \right)=1-\prod_{t<\tau }\left(1-\lambda_{i,t}\right).$

This survival head allowed the model to estimate cause‐specific absolute progression risk rather than only ranking participants. Because death was treated as censoring rather than as a competing event, absolute 5‐year risk estimates may be overestimated when mortality precludes observation of late AMD.

### 6.12 Counterfactual Protocol Alignment

Device identity and camera model were not used as prediction features. Protocol variables were used only for eligibility, quality control, sensitivity analysis, and drift audit. To reduce cohort/protocol shortcuts, ORBIT‐AMD used a gradient‐reversal cohort critic and a distributional alignment penalty on the image‐derived latent representation. Age and systemic‐risk variables were not used as adversarial targets; when included in downstream clinical‐context features, they remained available to the prediction heads. The adversarial critic used cohort/protocol labels as the nuisance target and did not use age, AMD stage, lesion‐concept labels or progression outcomes as adversarial targets. The alignment objective was therefore designed to reduce acquisition/protocol separability, but it cannot mathematically guarantee complete removal of nuisance style while preserving every biologically meaningful cohort difference. The maximum mean discrepancy (MMD) between UKB and TMUEH latent distributions was monitored as:

$$\mathrm{MM}D^{2}\left(P,Q\right)={∥\frac{1}{n}\sum_{i=1}^{n}\phi \left(h_{i}^{P}\right)-\frac{1}{m}\sum_{j=1}^{m}\phi \left(h_{j}^{Q}\right)∥}_{H}^{2}$$

where *P* and *Q* represent the two cohort distributions, and *ϕ(·)* maps embeddings into a reproducing‐kernel Hilbert space. The alignment coefficient increased linearly from 0 to 0.20 during the first 30 epochs.

### 6.13 Model Training and Optimization

The total model objective combined reconstruction, concept prediction, ordinal staging, survival prediction and protocol‐alignment losses:

$L=\lambda_{\mathrm{rec}}\;L_{\mathrm{rec}}+\lambda_{\mathrm{concept}}L_{\mathrm{concept}}+\lambda_{\mathrm{ord}}L_{\mathrm{ord}}+\lambda_{\mathrm{surv}}L_{\mathrm{surv}}+\lambda_{\mathrm{align}}L_{\mathrm{align}}.$

Weights were set to *λrec* = 1.0, *λconcept* = 0.7, *λord* = 1.0, *λsurv* = 1.0 and *λalign* = 0.2 after warm‐up. Models were trained for 100 epochs using AdamW, learning rate 3 × 10^−4^, weight decay 0.05, batch size 128, and cosine learning‐rate decay with 10 warm‐up epochs(Figures S2, S3 and Table S4). Early stopping used validation five‐year progression AUROC and ordinal kappa with patience of 15 epochs.

### 6.14 Baseline Models

ORBIT‐AMD was compared with five baselines: clinical‐only gradient boosting, CFP‐only convolutional neural network, OCT‐only convolutional neural network, bimodal late fusion and a RETFound‐linear‐style frozen foundation representation with a linear or shallow downstream head. All baselines used the same participant‐level splits and the same external test cohort.

### 6.15 Performance Evaluation

For prevalent late‐AMD detection, performance was assessed using AUROC, AUPRC, sensitivity, specificity, PPV, negative predictive value, Brier score, calibration intercept, calibration slope, and expected calibration error. Ordinal staging was evaluated using accuracy, macro‐F1, class‐specific recall, adjacent‐stage accuracy, and quadratic weighted kappa. Progression prediction was evaluated using time‐dependent AUROC, AUPRC, Harrell C‐index, Brier score, calibration curves, and risk‐quartile incidence (Table S11). Because the progression model used a cause‐specific survival formulation, absolute cumulative‐risk estimates were interpreted under the independent‐censoring assumption rather than as competing‐risk cumulative incidence (Table S14).

Calibration was summarized using expected calibration error:

$$\mathrm{ECE}\;=\sum_{b=1}^{B}\frac{n_{b}}{N}\;\left|{\hat{p}}_{b}-{\hat{o}}_{b}\right|,$$

where B = 10 calibration bins, *n_b_* is the number of participants in bin *b, p_b_* is the mean predicted risk and *o_b_* is observed event frequency.

### 6.16 Decision‐Curve and Workload Analysis

Clinical utility was assessed using decision‐curve analysis. At threshold *p_t_*, net benefit was calculated as:

$\mathrm{NB}\left(p_{t}\right)=\frac{\mathrm{TP}}{N}\;-\frac{\mathrm{FP}}{N}\cdot \frac{p_{t}}{1-p_{t}}.$

Thresholds were selected to illustrate two practical operating points: a UKB population‐imaging enhanced‐review threshold and a TMUEH external low‐workload review‐prioritization threshold. The TMUEH operating point was not intended to represent a high‐sensitivity screening threshold. Workload was defined as the proportion of baseline non‐late participants referred for enhanced review at the selected threshold.

### 6.17 Subgroup, Ablation and Robustness Analyses

Subgroup analyses were performed by sex, age group, hypertension status, diabetes status, and image‐quality quartile. For each subgroup, event counts and confidence intervals were reported where possible. Ablations removed one component at a time, including masked pretraining, bilateral graph attention, concept bottleneck, ordinal‐survival coupling and counterfactual alignment. Each ablated model was retrained using the same training and validation split before evaluation. Robustness analyses tested label‐noise perturbation, missing‐concept perturbation, reduced image quality, and local recalibration in the external cohort. These analyses were considered exploratory diagnostics rather than definitive evidence of deployment readiness.

### 6.18 Statistical Analysis

Continuous variables were summarized as mean with standard deviation (SD) or median with interquartile range (IQR), as appropriate, and categorical variables were summarized as counts and percentages. Baseline characteristics were compared between cohorts using standardized mean differences (SMDs), with an absolute SMD greater than 0.10 considered indicative of meaningful imbalance. All model development, validation, and internal testing were performed at the participant level to avoid inter‐eye information leakage.

For binary late‐AMD detection, discrimination was evaluated using AUROC and AUPRC with 95% confidence intervals (CIs) estimated by participant‐level bootstrap resampling for the primary ORBIT‐AMD model. Sensitivity, specificity, PPV, negative predictive value, accuracy, and F1 score were calculated at prespecified operating thresholds. For ordinal AMD staging, performance was assessed using overall accuracy, macro‐F1, class‐specific recall, adjacent‐stage accuracy, and quadratic weighted kappa. For 5‐year progression prediction among baseline non‐late participants, censoring‐aware time‐dependent AUROC, AUPRC, Harrell C‐index, and Brier score were reported under a cause‐specific discrete‐time survival framework. Calibration was evaluated using calibration plots, calibration slope, calibration intercept, and expected calibration error. Decision‐curve analysis was used to estimate net benefit across clinically plausible thresholds, and workload was defined as the proportion of participants referred for enhanced review. Baseline‐model and ablation‐model comparisons were interpreted as exploratory component diagnostics; when resampled prediction outputs were not available for all variants, point estimates were reported with this limitation stated in the table footnotes.

Subgroup analyses were performed by sex, age group, hypertension status, diabetes status, and image‐quality quartile. Robustness was assessed using component ablation, label‐noise perturbation, concept perturbation, reduced image‐quality testing, and local recalibration in the external cohort. Unless otherwise specified, two‐sided statistical tests were used, and P<0.05 was considered statistically significant. Because subgroup, ablation, and robustness analyses were exploratory, emphasis was placed on effect sizes, confidence intervals, and consistency rather than nominal statistical significance. All analyses were conducted using reproducible scripts with prespecified random seeds for data splitting and model initialization.

### 6.19 Ethics Approval and Informed Consent

This study was conducted in accordance with the Declaration of Helsinki. The TMUEH cohort was approved by the Medical Ethics Committee of Tianjin Medical University Eye Hospital (Approval No. 2024KY‐13), and all participants provided written informed consent.

## Author Contributions

**Xuehao Cui**: conceived and designed the study, performed statistical analyses, interpreted results, drafted the manuscript, and served as the first and corresponding author. **Dejia Wen**: contributed equally to data analysis, environmental exposure assessment, and manuscript drafting. **Patrick Yu‐Wai‐Man** and **Xiaorong Li**: supervised the overall study design, interpretation, and manuscript revision and served as co‐corresponding authors.

## Funding

This work was supported by the 2024 Independent and Open Research Project of Tianjin Key Laboratory of Retinal Functions and Diseases, Tianjin Medical University Eye Hospital (Hospital‐level Youth Project; No. 2024tjswmg‐004).

## Conflicts of Interest

The authors declare no conflicts of interest.

## Supporting information




**Supporting File 1**: advs76361‐sup‐0001‐TableS1‐S18.zip.

**Supporting File 2**: advs76361‐sup‐0002‐DataFile.zip.

## Data Availability

UK Biobank data are available to approved researchers through standard UK Biobank access procedures. The TMUEH dataset contains institutional clinical imaging and health‐record data and is not publicly available because of patient privacy, consent, and institutional governance restrictions. De‐identified aggregate data supporting the main results may be made available from the corresponding author upon reasonable request and institutional approval. To support transparent peer review and computational reproducibility, we provide a confidential reproducibility package as supplementary review material. The package includes data tables, executable analysis code, and reusable source‐code modules. The data tables include de‐identified tabular review data and aggregate manuscript result tables for verifying data cleaning, model‐output evaluation, calibration, decision‐curve analysis, subgroup summaries, ablation‐style summaries and manuscript‐style figure generation. The executable code and source‐code modules implement data integrity checking, preprocessing, participant‐level split auditing, leakage checking, metric calculation, ordinal‐stage assessment, cause‐specific progression‐risk evaluation, calibration, decision‐curve analysis, subgroup analysis, ablation‐style summaries, figure generation, protected‐environment image‐processing interfaces, image‐concept extraction interfaces, and local model‐training interfaces.
